# Bone Health in Metastatic Hormone-Sensitive Prostate Cancer: Where We Stand and Where We Can Improve

**DOI:** 10.3390/cancers17243977

**Published:** 2025-12-13

**Authors:** Juan Antonio Encarnación, Enrique López-Jiménez, Jose Luis Alonso-Romero, Paula Ruiz, Silverio Ros, Maria Isabel De la Fuente, Francisco López, Enrique Cárdenas, Ana Laborda, Marta Sánchez-Pérez, Cristina Rodríguez, Clara Manso, Nicolas Dario Ortega-López, Pedro López-Cubillana, Pablo Luis Guzman Martínez-Valls, Enrique Cao-Avellaneda, Pedro Ángel López-González, Alicia López-Abad

**Affiliations:** 1 Faculty of Medicine, University of Murcia, 30120 Murcia, Spain; juanantonio.encarnacion@um.es (J.A.E.); josel.alonso2@carm.es (J.L.A.-R.); misabell.fuente@carm.es (M.I.D.l.F.); enrique.cardenas@carm.es (E.C.); analabordasegovia@gmail.com (A.L.); sanchez.perez.marta@gmail.com (M.S.-P.); cristina.rodriguez15@carm.es (C.R.); 2 Department of Radiation Oncology, Virgen de la Arrixaca University Clinical Hospital, 30120 Murcia, Spain; franciscoj.lopez3@carm.es; 3 Murcian Institute for Biosanitary Research “Pascual Parrilla”, 30120 Murcia, Spain; paula.ruiz@carm.es (P.R.); silverio.ros@carm.es (S.R.); 4 Department of Medical Oncology, Virgen de la Arrixaca University Clinical Hospital, 30120 Murcia, Spain; 5 Department of Intensive Care Medicine, Virgen de la Arrixaca University Clinical Hospital, 30120 Murcia, Spain; clara92mm@hotmail.com; 6 Department of Internal Medicine, Virgen de la Arrixaca University Clinical Hospital, 30120 Murcia, Spain; darionick@yahoo.es; 7 Department of Urology, Virgen de la Arrixaca University Clinical Hospital, 30120 Murcia, Spain; pedro.lopez9@carm.es (P.L.-C.); pablol.guzman@carm.es (P.L.G.M.-V.); enrique.cao@carm.es (E.C.-A.); pedroa.lopez@carm.es (P.Á.L.-G.); alicialopezabad@gmail.com (A.L.-A.)

**Keywords:** androgen deprivation therapy, metastatic hormone-sensitive prostate cancer, bone health management, calcium and vitamin D, osteoporosis, denosumab, bone densitometry, antiresorptive agents, osteopenia

## Abstract

Background: Androgen deprivation therapy (ADT) is a fundamental component of treatment for metastatic hormone-sensitive prostate cancer (mHSPC), but it accelerates bone mineral density loss and increases fracture risk. International guidelines recommend calcium and vitamin D supplementation, baseline dual-energy X-ray absorptiometry (DXA), and antiresorptive therapy in patients with osteoporosis. Methods: We conducted a retrospective review of 156 mHSPC patients treated with ADT at a tertiary hospital between January 2022 and December 2024. We assessed adherence to guideline-recommended bone health measures. Collected variables included age, ADT duration, calcium/vitamin D supplementation, DXA testing, antiresorptive treatment, and fracture events. Exploratory stratified analyses were performed, and proportions were reported with 95% confidence intervals (CIs). Results: Calcium/vitamin D supplementation was prescribed in 50.6% of patients (95% CI: 42.9–58.4), baseline DXA was performed in 12.8% (95% CI: 8.5–18.9), and denosumab was administered in 5.1% of the cohort (95% CI: 2.6–9.8). The median follow-up was 23 months, with a fracture incidence of 0.67 events per 100 person-years. Stratified analyses showed lower adherence in older patients, those with prolonged ADT exposure, and those with high metastatic burden. Conclusions: Adherence to guideline-recommended bone health measures in patients with mHSPC receiving ADT was markedly suboptimal. These findings underscore the need to implement standardized institutional protocols to ensure systematic supplementation, routine DXA monitoring, and appropriate antiresorptive therapy.

## Simple Summary

Men with metastatic hormone-sensitive prostate cancer (mHSPC) treated with androgen deprivation therapy (ADT) are at high risk of osteoporosis and fractures, yet bone health prevention is often overlooked in clinical practice. In this study, we evaluated real-world adherence to guideline-recommended measures—including calcium and vitamin D supplementation, DXA screening, and antiresorptive therapy—in a consecutive cohort of patients with mHSPC. Our findings show that preventive strategies are implemented far less frequently than recommended, with low rates of supplementation, DXA testing, and treatment of osteoporosis. These results highlight an important evidence-to-practice gap and underscore the need for standardized institutional protocols to ensure systematic bone health preservation in patients undergoing ADT.

## 1. Introduction

Metastatic hormone-sensitive prostate cancer (mHSPC) represents a critical stage of the disease with a significant impact on both survival and patients’ quality of life. Androgen deprivation therapy (ADT) constitutes the cornerstone of treatment, either as monotherapy or in combination with agents targeting the androgen receptor signaling pathway or with chemotherapy [1,2,3,4,5,6]. However, ADT is consistently associated with metabolic and skeletal adverse effects, most notably the accelerated loss of bone mineral density and the consequent increased risk of fractures [7,8,9,10].

Several studies have demonstrated that prolonged ADT can induce up to a 10% reduction in bone mineral density during the first two years of treatment, resulting in a 1.5–2.0-fold higher relative risk of fracture compared with untreated men [11,12,13,14]. This risk is further exacerbated by characteristics inherent to the affected population, such as advanced age, frailty, and the presence of comorbidities.

Major international guidelines (EAU, NCCN, ASCO, and NOGG) [15,16,17,18,19] unanimously recommend implementing preventive measures from the initiation of therapy: universal supplementation with calcium and vitamin D, baseline and annual dual-energy X-ray absorptiometry (DEXA) scans, and the use of antiresorptive agents (denosumab or bisphosphonates) in patients with osteoporosis or high fracture risk. Despite the robustness of these recommendations, multiple real-world studies have consistently demonstrated low implementation rates, suggesting the existence of a relevant gap between scientific evidence and routine clinical practice [20,21].

Furthermore, population-based registries and multicenter studies have shown that adherence to clinical guideline recommendations in this area remains suboptimal. In international cohorts, fewer than 40% of patients receiving ADT are adequately supplemented with calcium and vitamin D, and baseline bone mineral density testing is performed in only about 10–20% of cases [22]. These findings highlight the need to establish standardized bone prevention protocols in clinical practice. Assessing the magnitude of this gap in local contexts such as ours is essential to designing improvement strategies and ensuring that the benefits of oncologic therapies are not compromised by preventable skeletal complications.

The clinical impact of fractures in this patient population is particularly relevant. Several studies have shown that men with prostate cancer undergoing ADT have higher mortality rates following hip fractures compared to the general population, as well as significant deterioration in quality of life and functional autonomy [23,24]. A meta-analysis reported a 13% improvement in overall survival among mHSPC patients treated with bisphosphonates [25]. Additionally, osteoporosis and osteopenia frequently remain underdiagnosed, contributing to the underuse of antiresorptive therapies with proven efficacy in reducing fracture risk.

Androgen deprivation therapy induces profound skeletal alterations through well-described molecular and cellular pathways. The sharp decline in circulating testosterone and estradiol reduces osteoblastic activity and shifts bone remodeling toward enhanced osteoclastic resorption by increasing the RANKL/OPG ratio. ADT also promotes systemic inflammation, decreases IGF-1 levels, and contributes to sarcopenia and increased adiposity, all of which further impair bone microarchitecture and strength. Bisphosphonates counteract these mechanisms by binding to hydroxyapatite surfaces and inhibiting farnesyl pyrophosphate synthase within the mevalonate pathway of osteoclasts, ultimately inducing osteoclast apoptosis and reducing bone resorption. These biological effects justify their preventive and therapeutic value in patients undergoing prolonged hormonal suppression.

Several patient-related and treatment-related factors further increase the risk of ADT-induced bone loss. Chronic glucocorticoid use, commonly encountered in patients receiving concomitant therapies, exacerbates bone resorption and reduces bone formation. Additional determinants include low body mass index, baseline vitamin D deficiency, smoking, frailty, and sarcopenia, all of which are prevalent in this population and potentiate fracture risk. These factors underscore the need for systematic assessment and early implementation of preventive strategies.

In this context, our objective was to analyze the degree of adherence to international recommendations for the prevention of bone toxicity in a cohort of patients with mHSPC treated with ADT at a tertiary hospital in the Region of Murcia, Spain.

## 2. Materials and Methods

### 2.1. Study Design and Patients

A retrospective, descriptive study was conducted at the Virgen de la Arrixaca University Clinical Hospital (Murcia, Spain). The study population included 156 patients diagnosed with metastatic hormone-sensitive prostate cancer (mHSPC) who received androgen deprivation therapy (ADT) between January 2022 and December 2024, with the aim of identifying the magnitude of the gap between scientific evidence and routine clinical practice.

#### 2.1.1. Inclusion Criteria

Histologically confirmed diagnosis of prostate adenocarcinoma.

Hormone-sensitive metastatic disease was defined by the presence of bone and/or visceral metastases in the setting of non-castrate serum testosterone levels.

Initiation of ADT was conducted during the study period regardless of concomitant use of androgen receptor pathway inhibitors or chemotherapy.

Availability of complete electronic medical records documenting treatment was ensured and a minimum follow-up of 6 months.

#### 2.1.2. Exclusion Criteria

Incomplete clinical records regarding ADT exposure or follow-up.

Early discontinuation or permanent withdrawal of ADT for reasons unrelated to oncologic progression led to participant exclusion.

Patients with a prior diagnosis of osteoporosis receiving active treatment before ADT initiation (to avoid indication bias) were excluded.

#### 2.1.3. Definition of Bone Prevention

Adequate bone prevention was defined as the implementation of at least one of the prophylactic measures recommended by international guidelines (EAU, NCCN, ASCO, NOGG):

Calcium and vitamin D supplementation, regardless of baseline bone mineral density;

Baseline or follow-up dual-energy X-ray absorptiometry (DEXA);

Antiresorptive therapy (denosumab, Prolia^®^™, developed by Amgen, Thousand Oaks, CA, USA) in the presence of osteoporosis (T-score ≤ −2.5 or documented fragility fracture).

For the descriptive analysis, partial bone prevention was defined as the implementation of only one preventive measure, whereas complete bone prevention required all three (supplementation, DEXA, and antiresorptive therapy when indicated).

### 2.2. Data Collection

Clinical data were extracted from electronic medical records in August 2025. The following variables were collected:

Preventive bone supplementation: administration of calcium and vitamin D.

Bone health monitoring: baseline and follow-up DEXA scans.

Antiresorptive therapy: administration of denosumab (Prolia^®^) in patients with osteoporosis, defined as a T-score ≤ −2.5 or a history of fragility fracture.

Additional variables: patient age, duration of ADT, use of antidepressants, and occurrence of osteoporotic fractures during follow-up.

All data were anonymized prior to analysis, ensuring confidentiality in accordance with current regulations (Regulation [EU] 2016/679 of the European Parliament and of the Council, and Organic Law 3/2018 on the Protection of Personal Data and Guarantee of Digital Rights).

The study was conducted in accordance with the ethical principles of the Declaration of Helsinki and Good Clinical Practice (ICH-GCP). The study protocol was reviewed and approved by the Clinical Research Ethics Committee of the Virgen de la Arrixaca University Clinical Hospital (Murcia, Spain).

### 2.3. Objectives

The primary objective was to assess adherence to international guideline recommendations (EAU, NCCN, ASCO, NOGG) regarding bone health management in patients treated with ADT. Specifically, the study evaluated the proportion of patients who received calcium/vitamin D supplementation, underwent DEXA screening, and received denosumab therapy when indicated.

### 2.4. Statistical Analysis

A descriptive statistical analysis was performed. Continuous variables were summarized as mean ± standard deviation (SD) or median and interquartile range (IQR), depending on distribution. Categorical variables were reported as frequencies and percentages with 95% confidence intervals (CIs). Comparisons between subgroups (age groups, ADT duration, metastatic burden, and co-treatments) were performed using chi-square or Fisher’s exact tests for categorical variables and *t*-tests or Mann–Whitney U tests for continuous variables. Incidence rates of fractures were calculated per 100 person-years. An exploratory binary logistic regression model was conducted to identify predictors of inadequate bone health management, defined as lack of supplementation and absence of DXA screening. Variables included age, ADT duration (<24 vs. ≥24 months), metastatic burden (low vs. high), and use of androgen receptor signaling inhibitors. Statistical significance was set at *p* < 0.05. Analyses were conducted using IBM SPSS Statistics version 25.0 (IBM Corp., Armonk, NY, USA).

## 3. Results

### 3.1. Patient Characteristics

A total of 156 patients with metastatic hormone-sensitive prostate cancer (mHSPC) treated with androgen deprivation therapy (ADT) were included in the study. The mean age of the cohort was 74.2 ± 8.2 years (range, 55–93). The mean duration of ADT at the time of analysis was 37.3 ± 19.2 months.

### 3.2. Bone Health Preventive Measures

**Figure 1:**
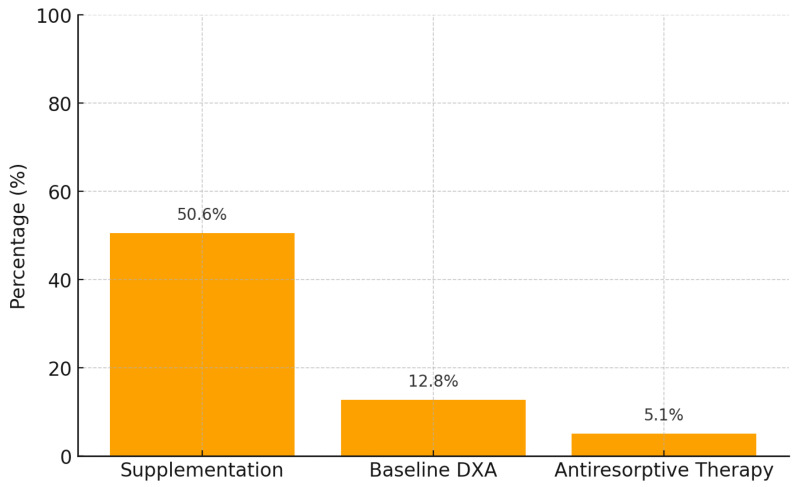
Adherence gaps in bone health management.

Of the total cohort, 79 patients (50.6%) received calcium and vitamin D supplementation. Baseline dual-energy X-ray absorptiometry (DEXA) was performed in 20 patients (12.8%), and 8 patients (5.1%) with osteoporosis were treated with denosumab (Prolia^®^), as shown in Figure 1.

Only two patients (1.3%) experienced fragility fractures during follow-up; neither had received any preventive measures.

The median follow-up of the cohort was 23 months (range 6–39 months). Based on the median follow-up, the estimated incidence of fragility fractures was approximately 0.67 events per 100 person-years. Both documented fractures occurred in patients who had not received supplementation, DXA evaluation, or antiresorptive therapy, reinforcing the clinical relevance of systematic preventive strategies.

### 3.3. Comparison with International Guidelines

There is a notable discrepancy between international guidelines (EAU, NCCN, ASCO, NOGG) and our patients. While guidelines mandate universal Calcium and Vitamin D supplementation and baseline DEXA scans, clinical practice shows adherence rates of only 50.6% and 12.8%, respectively. Furthermore, antiresorptive therapy with Denosumab is severely underutilized (5.1%), despite its indication for all patients with osteoporosis. Given the mean patient age of 74 years and prolonged ADT exposure (averaging 37 months), this gap in preventive care highlights a significant risk for treatment-induced bone loss and fragility fractures. This can be seen in Table 1.

### 3.4. Stratified Analyses

Age stratification did not reveal relevant differences in supplementation rates or DXA assessment. Patients aged ≥80 years showed similar rates of supplementation and DXA evaluation compared with those younger than 70 years. Likewise, patients with ADT duration ≥24 months did not demonstrate a lower likelihood of having undergone baseline DXA compared with those treated for less than 24 months. No significant differences in adherence to preventive measures were observed according to metastatic burden (high vs. low volume) or concomitant use of androgen receptor signaling inhibitors, indicating that treatment intensification was not associated with improved bone health management. Overall, the exploratory analyses did not identify clinical subgroups with differential adherence patterns.

### 3.5. Exploratory Logistic Regression

An exploratory logistic regression analysis was performed to evaluate potential predictors of inadequate bone health management. Age category, ADT duration, metastatic burden, and use of androgen receptor signaling inhibitors were included in the model. None of these variables demonstrated a statistically significant association with inadequate preventive care (all *p* > 0.05). These findings indicate that the low adherence observed in the cohort was uniform across clinical subgroups, suggesting that system-level factors rather than patient-specific characteristics are likely responsible for the gaps identified.

## 4. Discussion

In this cohort of 156 patients with metastatic hormone-sensitive prostate cancer (mHSPC) receiving androgen deprivation therapy (ADT), adherence to guideline-recommended bone health measures was low. Only half of the patients received calcium and vitamin D supplementation, 12.8% underwent baseline DXA assessment, and 5.1% received antiresorptive therapy. These findings confirm that there is a substantial evidence–practice gap in the prevention of ADT-induced skeletal toxicity. While antiresorptive therapy is indicated only for patients with osteoporosis, the low rate of DXA scanning limited the ability to adequately identify candidates for treatment [22,23,24].

Our results align with those from international cohorts consistently reporting suboptimal adherence to bone health recommendations in men undergoing ADT. North American and European studies have documented DXA assessment rates typically below 20–25% and supplementation rates commonly below 40–50% [20,22,23,24]. More recently, a population-based analysis in the United Kingdom and Central Europe also confirmed persistently low rates of bone mineral density screening and prophylactic supplementation in this setting [26]. The present findings therefore reinforce that inadequate implementation of guideline-recommended bone health measures remains a widespread and unresolved issue.

The clinical relevance of this gap is well established. Men receiving ADT experience accelerated bone mineral density loss and an increased risk of fragility fractures [11,27]. Hip fractures in this population are associated with substantial morbidity, functional decline, and one-year mortality rates ranging from 14% to 36% [28,29]. The risk of death is highest during the first months after fracture and remains elevated compared with the general population [30]. Denosumab has demonstrated significant reductions in bone mineral density loss and vertebral fracture risk in men receiving ADT [26,31], supporting the need for timely identification and treatment of high-risk patients.

In our cohort, low adherence to supplementation, DXA screening, and antiresorptive therapy was observed across age groups, metastatic burden categories, and treatment-intensity levels, indicating that no specific clinical subgroup received better preventive care. These findings suggest that the gap is systemic rather than patient-dependent. The growing use of treatment-intensification strategies with androgen receptor signaling inhibitors (ARSIs), which prolong survival [32,33,34,35], further increases cumulative exposure to ADT and reinforces the need for structured bone health management integrated into routine oncologic care.

Several factors likely contribute to the observed deficiencies, including underestimation of fracture risk in men, prioritization of oncologic treatment over supportive measures, absence of standardized institutional pathways, and lack of automated alerts in electronic prescribing systems. Organizational limitations that hinder universal baseline DXA assessment may further exacerbate these disparities [22,23,24].

This study has limitations inherent to its retrospective, single-center design, including potential missing data and incomplete documentation of DXA examinations performed outside the institutional network. Actual adherence to supplementation or antiresorptive therapy could not be fully assessed. Although follow-up duration varied across patients, fragility fractures occurred exclusively in individuals who had not received any preventive measures.

Despite these limitations, the findings emphasize the urgent need for structured interventions to improve bone health management in mHSPC. Practical steps include standardized baseline and periodic DXA scanning, universal supplementation at ADT initiation supported by electronic reminders, and streamlined access to antiresorptive therapy for patients with osteoporosis. Future quality-improvement initiatives should incorporate both process indicators (e.g., supplementation and DXA rates) and outcome indicators such as fracture incidence and changes in bone mineral density at 12–24 months.

Moreover, although preventive measures such as calcium/vitamin D supplementation, DXA monitoring, and antiresorptive therapy are known to mitigate ADT-induced bone loss, our retrospective dataset did not include longitudinal bone mineral density measurements. As a result, we were unable to determine whether patients who received preventive measures experienced less bone loss or a delayed onset of osteoporosis compared with those who did not. Similarly, overall survival could not be assessed, as survival data were not uniformly available and oncologic outcomes were outside the scope of this analysis. Consequently, we could not compare survival between patients with and without preventive interventions. Given the established association between fragility fractures, functional decline, and increased mortality in men receiving ADT, future prospective studies incorporating systematic DXA follow-up and survival endpoints are needed to clarify the clinical impact of optimized bone health management.

## 5. Conclusions

In this cohort of patients with metastatic hormone-sensitive prostate cancer treated with androgen deprivation therapy, a significant gap was observed between international guideline recommendations and routine clinical practice in the prevention of bone loss. Calcium and vitamin D supplementation was prescribed in only half of the patients; fewer than 15% underwent baseline DEXA assessment; and the use of antiresorptive therapy in cases of osteoporosis was markedly limited. These findings underscore the need to optimize the implementation of bone prevention strategies as an integral component of the management of this patient population.

## Figures and Tables

**Table 1:** Comparison between real-world clinical practice and international guideline recommendations for bone health prevention in patients with prostate cancer receiving androgen deprivation therapy (ADT).

Preventive/Therapeutic Measure	Clinical Practice	Guideline Recommendation (EAU, NCCN, ASCO, NOGG)
Calcium + Vitamin D supplementation	79 (50.6%)	100% of patients on ADT should receive supplementation, regardless of baseline bone mineral density (BMD).
Baseline and follow-up DEXA scan	20 (12.8%)	Baseline DEXA scan mandatory before initiating ADT; repeat every 1–2 years.
Antiresorptive therapy (Denosumab/Prolia^®^) in osteoporosis	8 (5.1%)	Indicated in all patients with osteoporosis (T-score ≤ −2.5).
Documented fragility fractures	N = 2	High incidence expected without prevention; active prevention strongly recommended.
Mean age	74.2 ± 8.2 years	Advanced age is a major risk factor requiring systematic preventive measures.
Mean ADT duration	37.3 ± 19.2 months	Risk of bone loss increases after 6–12 months of ADT and accumulates with treatment duration.

## Data Availability

The data supporting the findings of this study are available from the corresponding author upon reasonable request. Restrictions apply because the dataset contains patient-level clinical information protected by institutional privacy policies.
